# Associated factors for in-hospital mortality in patients with fulminant myocarditis: a systematic review and meta-analysis

**DOI:** 10.3389/fcvm.2026.1938794

**Published:** 2026-09-28

**Authors:** Haojie Zhou, Qiang Zhang, Xi Zheng, Guifeng Zhao, Ran Li

**Affiliations:** 1 School of Nursing, Dali University, Yunnan, China; 2 Department of Cardiovascular Medicine, The Second Affiliated Hospital of Chongqing Medical University, Chongqing, China

**Keywords:** associated factors, fulminant myocarditis, in-hospital mortality, meta-analysis, systematic review

## Abstract

**Background:**

Fulminant myocarditis (FM) progresses rapidly and carries high all-cause in-hospital mortality. Patients in the early phase often develop cardiogenic shock and malignant ventricular arrhythmias, with widely varying mortality rates reported across studies. Existing single-center cohorts yield conflicting prognostic findings with marked cross-cohort heterogeneity, and no standardized meta-analysis with GRADE certainty grading has been published to guide risk stratification. This meta-analysis pooled prognostic associations of all-cause in-hospital mortality in FM to inform acute risk evaluation and personalized care.

**Methods:**

Eleven Chinese and English electronic databases were searched from database establishment through June 2025. Eligible studies were observational cohorts reporting prognostic associations of all-cause in-hospital mortality among FM patients. Two independent reviewers completed screening, data extraction and quality assessment separately. The Newcastle-Ottawa Scale (NOS) and AHRQ checklist were adopted to evaluate methodological quality. Pooled analyses were performed via the statsmodels package in Python, and the GRADE framework was applied to rate the certainty of evidence.

**Results:**

26 retrospective cohort studies with 4,890 FM patients were enrolled. Ten potential factors were meta-analyzed, of which six showed statistically significant associations with in-hospital mortality (*P* < 0.05), including age (OR = 1.03, 95% CI: 1.02–1.04), cardiac arrest or CPR (OR = 2.57, 95% CI: 1.49–4.44), lactate (OR = 1.08, 95% CI: 1.00–1.16), cTnI (OR = 1.04, 95% CI: 1.01–1.07), LVEF (OR 0.94, 95% CI 0.90–0.97), and intravenous immunoglobulin therapy (OR = 0.12, 95% CI: 0.02–0.86). CK-MB, serum creatinine, total bilirubin, and VT/VF showed no statistically significant association (all *P* > 0.05). GRADE evidence ratings indicated low-certainty evidence for age and cTnI, and very-low-certainty evidence for the remaining significant factors. Sensitivity analyses indicated unstable pooled estimates for lactate and intravenous immunoglobulin therapy.

**Conclusions:**

Four factors showed positive associations with elevated all-cause in-hospital mortality. LVEF was inversely associated with mortality, whereas the inverse association for IVIG represents a fragile treatment-related signal. These findings may inform acute risk screening, but most associations were supported by low or very low certainty evidence and should not be interpreted as causal. All included data were derived from retrospective cohorts. Standardized multicenter prospective studies are required to verify these prognostic associations.

**Systematic Review Registration:**

https://www.crd.york.ac.uk/PROSPERO/view/CRD420261399446, identifier CRD420261399446.

## 1 Introduction

Fulminant myocarditis (FM) is the most severe and rapidly progressive subtype within the myocarditis spectrum (1), defined by severe inflammatory myocardial damage (2) and acute heart failure that can quickly deteriorate into cardiogenic shock and malignant arrhythmias (3). It represents a critical cardiovascular emergency associated with substantial fatality burden among young and middle-aged adults (4). Compared with acute non-fulminant myocarditis, FM presents with more profound initial hemodynamic compromise (5). Notably, patients who receive timely circulatory support and immunomodulatory therapy can achieve complete cardiac recovery with favorable long-term outcomes, which reflects the marked clinical and prognostic heterogeneity of FM. This inherent interindividual variability largely explains the widely variable in-hospital mortality rates reported in previous studies, ranging from 3.7% to 55.6% (6, 7). Such discrepancies are primarily attributable to differences in baseline patient characteristics, mechanical circulatory support utilization rates, timing of therapeutic intervention, and case selection criteria across studies. This substantial between-study variation highlights the lack of a unified, validated risk stratification tool for FM, hindering accurate early identification of high-risk patients.

Current clinical guidelines have established standardized management regimens for FM, prioritizing hemodynamic support, immunomodulatory therapy, and symptomatic care, which has substantially improved overall patient outcomes (8). Nevertheless, a subset of patients still experiences progressive, irreversible clinical deterioration and in-hospital mortality despite adherence to guideline-concordant therapy. This dilemma indicates that generalized, one-size-fits-all therapeutic strategies fail to fully accommodate the heterogeneous disease severity and prognostic diversity of FM. The absence of well-validated prognostic correlates for mortality severely limits the implementation of individualized acute-phase precision care and impedes further reductions in in-hospital mortality. In this context, systematically synthesizing clinical, laboratory, and therapeutic factors associated with in-hospital mortality in FM can facilitate efficient high-risk patient screening, individualized adjustment of treatment intensity, rational allocation of critical care resources, and improved short-term survival.

Numerous clinical investigations have explored potential prognostic associations factors for in-hospital mortality among patients with FM (9), covering demographic characteristics, acute clinical manifestations, biomarkers of myocardial injury and multi-organ dysfunction (10), and intensive care interventions (11). However, these single-center studies vary substantially in sample size, adopt inconsistent strategies for baseline confounder adjustment, and apply heterogeneous clinical endpoint definitions and statistical reporting formats. Accordingly, the direction and magnitude of the associations between candidate variables and all-cause in-hospital mortality remain inconsistent across cohorts, with conflicting findings commonly reported. Currently, there remains no consensus regarding whether core clinical markers, including age (12), cardiac troponin I (cTnI) (13), serum lactate (10), and left ventricular ejection fraction (LVEF) (14), are independent prognostic correlates of in-hospital mortality in FM. Most available evidence is derived from small-sample, single-center retrospective cohorts (14), which are prone to selection bias and insufficient statistical power. These limitations preclude reliable quantification of true prognostic associations and hinder the development of validated risk stratification systems for severe FM.

Systematic reviews and meta-analyses represent rigorous evidence synthesis approaches that aggregate quantitative data from independent cohorts, enhance statistical power, quantify between-study heterogeneity, and minimize random errors of individual primary studies, thereby helping to clarify clinical controversies and improve the transparency of evidence synthesis (15). Currently, no comprehensive quantitative meta-analysis has synthesized multidimensional factors for in-hospital mortality in FM through standardized effect-size pooling and formal evidence quality grading. This critical evidence gap leaves clinicians lacking quantitative, actionable evidence to refine risk stratification tools and optimize acute-phase therapeutic strategies. To address this deficiency, the present study systematically retrieved eligible original studies concerning in-hospital mortality among FM patients across global electronic databases. This rigorous systematic review and meta-analysis quantitatively synthesized variables significantly associated with in-hospital mortality, quantified the magnitude of each prognostic association, and evaluated the certainty of evidence using the GRADE (Grading of Recommendations, Assessment, Development and Evaluations) framework. This study aims to provide a comprehensive and certainty-graded synthesis to support early risk screening, individualized prognostic evaluation, and future prospective validation in high-risk patients with acute-phase FM.

## 2 Materials and methods

### 2.1 Study protocol and registration

This systematic review and meta-analysis was performed and reported in strict compliance with the Preferred Reporting Items for Systematic Reviews and Meta-Analyses (PRISMA) 2020 reporting guideline (16). A prospective study protocol was formally registered on the PROSPERO international prospective register of systematic reviews prior to data extraction and analysis, with the registration number CRD420261399446.

### 2.2 Search strategy

A comprehensive literature search was performed across eleven electronic databases, including PubMed, Web of Science, Cochrane Library, EBSCOhost, Wiley Online Library, Ovid, American Heart Association (AHA) Journals, China National Knowledge Infrastructure (CNKI), Wanfang Data, SinoMed, and VIP Chinese Journal Database. The search timeframe extended from the inception of each database to June 2025. A hybrid search strategy combining exploded Medical Subject Headings (MeSH) terms and customized free-text terms was adopted to maximize literature coverage. Search terms were categorized into three groups focusing on FM, in-hospital mortality, and risk predictors. Terms within each group were combined using the Boolean operator “OR” to expand retrieval scope, while “AND” was applied to integrate the three groups for targeted screening. The detailed search terms included “Fulminant Myocarditis”, “Acute Fulminant Myocarditis”, “Severe Myocarditis”, “Myocarditis”, “In-hospital Mortality”, “Hospital Mortality”, “Death”, “Risk Factors”, and “Predictors”, and other related terms. The full search strategy for PubMed is presented in Supplementary Table S1, and corresponding search strings for other databases were adaptively modified according to their respective retrieval syntax rules. Furthermore, the reference lists of all eligible original studies and relevant systematic reviews were manually screened to identify potentially missed qualified studies.

### 2.3 Inclusion and exclusion criteria

Inclusion criteria were as follows: (1) Observational studies including cohort, cross-sectional, or case-control designs. Randomized controlled trials were excluded because random assignment is ethically infeasible for patients with FM. (2) Participants were hospitalized patients diagnosed with FM following diagnostic criteria defined by each original study. Although diagnostic cut-offs differed across included cohorts, all enrolled patients exhibited the core phenotype of rapidly progressive acute myocardial inflammation accompanied by early-onset hemodynamic instability. (3) The primary endpoint was in-hospital all-cause mortality. Eligible studies needed to report mortality-related candidate factors and provide corresponding effect estimates (odds ratio, hazard ratio or risk ratio) with 95% confidence intervals (CIs). Multivariable-adjusted effect sizes were preferentially extracted. When raw datasets were available without reported adjusted outcomes, effect sizes were recalculated based on original study data. Studies lacking usable data following author correspondence were excluded from analysis. Only models adjusting for at least age and hemodynamic status were considered acceptable. (4) Articles were published in English or Chinese.

Exclusion criteria were as follows: (1) Systematic reviews, meta-analyses, case reports, commentaries, letters, animal experiments and laboratory-based studies. (2) Studies focusing on post-discharge long-term mortality or composite endpoints rather than in-hospital mortality. (3) Studies with insufficient data to calculate reliable effect-size estimates even after contacting authors. (4) Duplicate publications.

### 2.4 Literature screening and data extraction

All retrieved literatures were imported into Zotero 9.0.6 to remove duplicates. Two reviewers with systematic evidence-based training independently carried out literature screening in two phases according to pre-specified inclusion-exclusion criteria. Obvious irrelevant studies were excluded after screening titles and abstracts, and the remaining studies underwent full-text evaluation subsequently. Disagreements between the two investigators were resolved by group discussion. Consensus-unresolved conflicts were settled by a third senior reviewer.

Before formal data extraction, a pilot extraction covering 10 eligible articles was performed to standardize extraction rules. Two researchers independently extracted information via a self-designed standardized form. Extracted data included first-author name, publication year, study location, study design, overall sample size, participants' age, potential mortality-associated factors, adjusted confounders, effect-size indicators, corresponding 95% CIs and *P*-values. Mutual cross-verification was conducted after data-extraction finished.

### 2.5 Methodological quality assessment

The Newcastle-Ottawa Scale (NOS) was adopted to evaluate the methodological quality of cohort and case-control studies (17). This scale consists of eight evaluation items across three domains, including selection of participants (4 points), comparability between study groups (maximum of 2 points), and ascertainment of exposures or outcomes (3 points), with a maximum total score of nine points. Consistent with cut-off values widely adopted in prior meta-analyses, scores of 7-9, 5-6 and 0-4 were defined as high-quality, moderate-quality and low-quality studies, respectively. The checklist issued by the Agency for Healthcare Research and Quality (AHRQ) was used to assess cross-sectional studies (18). This checklist contained 11 evaluation items. One point was assigned for “yes” responses, zero points for “no” or “unclear” answers, whereas items marked “not applicable” were excluded from scoring. Scores of 8-11, 4-7 and 0-3 indicated high-quality, moderate-quality and low-quality cross-sectional studies correspondingly.

Two researchers independently carried out quality assessment. When disagreements occurred, the investigators first reached a consensus through thorough discussion. If conflicts remained unresolved, a senior reviewer made the final decision. According to our pre-registered protocol, studies categorized as low-quality by either the NOS or AHRQ checklist were excluded from subsequent pooled-analysis to minimize bias originating from flawed study design.

### 2.6 Statistical analysis

All statistical analyses were conducted in Python (version 3.10.4) using the statsmodels package. Pooled effect sizes were presented as odds ratios (ORs) together with corresponding 95% CIs. Between-study heterogeneity was assessed by the *Q*-test and *I*^2^ statistic. Heterogeneity was categorized as low (*I*^2^ ≤ 25%), moderate (25% < *I*^2^ ≤ 50%), and high (*I*^2^ > 50%). A fixed-effect model was applied when *I*^2^ < 50% and the *P*-value of *Q*-test exceeded 0.10. Otherwise, the random-effect model with the DerSimonian-Laird estimator was selected to calculate between-study variance. Quantitative meta-analysis was performed only when three or more studies provided available extractable effect-size data for a single candidate factor (19). All statistical tests were two-sided, and *P*-values less than 0.05 were defined as statistically significant.

#### 2.6.1 Pre-specified subgroup analyses

Pre-specified exploratory subgroup analyses were designed on the basis of clinically meaningful factors from previous studies regarding FM, covering age (pediatric vs. adult populations), geographic region (China vs. non-China populations), and acute-phase ECMO support (with vs. without routine use). Pooled results within a subgroup were generated if two or more studies belonged to that subgroup, while subgroup-level analysis would only be carried out for candidate factors with at least three overall included studies to avoid insufficient statistical power. Findings from subgroup analyses were interpreted as exploratory rather than conclusive evidence.

#### 2.6.2 Sensitivity analysis criteria

Leave-one-out sensitivity analysis was implemented by sequentially omitting one single study and recalculating pooled effect sizes from remaining included studies (20). The direction of pooled effects and statistical significance serve to evaluate the robustness of meta-analytic findings. The pooled estimate was considered robust if the effect direction stayed unchanged and the pooled *P*-value remained less than 0.05 after excluding each study. When removing any study reversed effect direction or made the pooled result statistically non-significant, the corresponding pooled outcome was viewed as unstable and heavily driven by that study.

#### 2.6.3 Publication bias assessment criteria

Funnel plots were generated to visually evaluate publication bias for factors pooled from three or more studies (21). Egger's linear regression test and Begg's rank-correlation test were performed only when ten or more studies were available to guarantee sufficient statistical power. For factors with fewer than ten included studies, publication bias was assessed only via funnel-plot scatter patterns given inadequate statistical performance of formal statistical tests. Marked asymmetry among funnel-plot points implied potential publication bias, whereas symmetric distribution indicated a low risk of publication bias. Significant publication bias was identified if the *P*-value fell below 0.05 for either Egger's test or Begg's test. Visual evaluation of funnel-plot results was subjective for analyses with a small number of eligible studies.

### 2.7 Evidence-certainty grading

The GRADE framework was adopted to assess the certainty of evidence for pooled outcomes (22). Given that all included studies belonged to observational designs, the baseline evidence certainty was initially rated as low. Evidence could be further downgraded by one or two levels when serious limitations existed regarding risk of bias, inconsistency, indirectness, imprecision and publication bias. Potential criteria for upgrading the certainty of observational evidence were predefined, including substantial effect magnitude, identifiable dose-response gradients, and the presence of plausible confounders that would solely attenuate the true effect. Following comprehensive evaluation, four distinct tiers of overall evidence certainty were defined, namely high, moderate, low, and very low. Two researchers independently performed GRADE evaluation. Disagreements were resolved through discussion or judged by a third senior reviewer if consensus could not be achieved. Only factors yielding pooled meta-analytic results received GRADE assessment, whereas subgroup-analysis outcomes and non-pooled factors were not graded.

## 3 Results

### 3.1 Literature screening results

The initial search retrieved 4,814 records in Chinese and English. After deduplication, 3,512 records remained. Title and abstract screening excluded clearly irrelevant studies, leaving 68 articles for full-text review. Following full-text screening against predefined eligibility criteria, 26 studies were ultimately included (6 in Chinese and 20 in English), all of which were retrospective cohort studies. Exclusions covered an ineligible study population (*n* = 11), unavailable full texts (*n* = 5), outcomes inconsistent with the predefined eligibility criteria (*n* = 17), incompatible study setting (*n* = 1), review articles (*n* = 3), and inappropriate study design (*n* = 5). The literature screening flow is presented in Figure 1.

**Figure 1:**
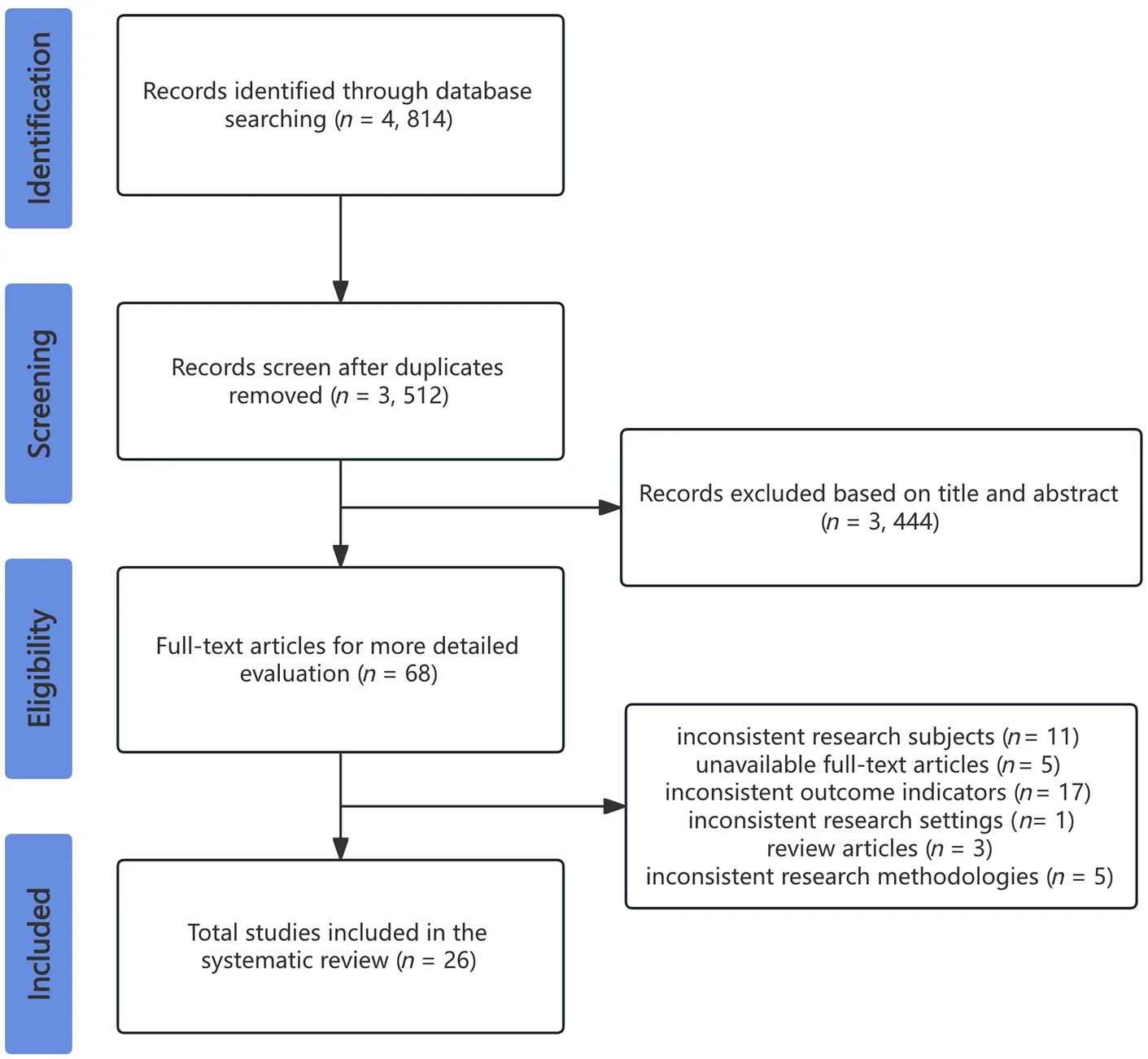
PRISMA flow diagram for literature screening and selection of included studies.

### 3.2 Characteristics of included studies

The basic characteristics of the 26 included studies (9, 13, 23–46) are summarized in Table 1. The studies originated from China (including Taiwan), Japan, South Korea, India, and several European countries, with publication years spanning 2015–2026. Sample sizes ranged from 24 to 344 patients, and study populations included both children and adults. All included studies were retrospective cohort designs.

**Table 1:** Baseline characteristics of the 26 included retrospective cohort studies.

First author	Publication year	Region	Age range	Simple	Single/multi-center	Study design	ECMO application	Quality assessment score	Quality grade	Candidate factors reported
Hao (23)	2023	China	≥18 years (median: 38 years)	202	Multi-center	Retrospective cohort study	Yes	8	High	(1), (2), (3), (4)
Hou (24)	2023	China	≥18 years (mean: 31.2 ± 11.3 years)	55	Single-center	Retrospective cohort study	Yes	8	High	(5), (6), (7), (8), (9)
Jayashree (25)	2021	India	1 month–12 years (median: 4 months)	120	Single-center	Retrospective cohort study	No	8	High	(10), (8)
Kanaoka (26)	2022	Japan	≥16 years (median: 54 years)	344	Multi-center	Retrospective cohort study	Yes	8	High	(1), (11), (12), (13), (14)
Kuo (27)	2023	Taiwan	≥18 years	33	Single-center	Retrospective cohort study	Yes	8	High	(15), (16), (17), (18)
Lee (28)	2020	Taiwan	1 month∼18 years	33	Multi-center	Retrospective cohort study	Partial	8	High	(12), (19)
Lee (29)	2021	South Korea	Pediatric: median 4 years; Adult: median 41.5 years	100	Single-center	Retrospective cohort study	Partial	6	Middle	(2), (20)
Liu (13)	2021	China	Median 35 years (adult patients)	220	Single-center	Retrospective cohort study	Partial	7	High	(20), (21), (22), (23), (24), (25)
Liu (30)	2021	China	Median 37 years (≥16 years old)	83	Single-center	Retrospective cohort study	Partial	7	High	(8), (13), (26), (27), (28)
Majunke (31)	2025	Multiple European countries	Median 43 years (≥18 years old)	271	Multi-center	Retrospective cohort study	Partial	6	Middle	(1), (12), (13), (23), (27), (29), (30), (31), (32), (33), (34), (35), (36), (37)
Miao (32)	2023	China	≥18 years, mean 42 ± 14 years	96	Single-center	Retrospective cohort study	Partial	7	High	(12), (13), (21), (38), (39), (40), (41), (42), (43)
Sawada (33)	2026	Japan	≥16 years, median 54 years	337	Multicenter	Retrospective cohort study	Partial	9	High	(1), (11), (12), (13), (20), (32), (35), (36), (44), (45)
Sun (9)	2022	China	4 months∼168 months, median 96 months	41	Single-center	Retrospective cohort study	Yes	6	Middle	(20), (27), (46)
Xie (34)	2022	China	Mean 8.35 ± 4.36 years (pediatric)	37	Multicenter	Retrospective cohort study	Partial	6	Middle	(12), (47)
Xu (35)	2018	China	Adult (survivor: 42.49 ± 17.72 years; non-survivor: 52.90 ± 10.62 years)	73	Single-center	Retrospective cohort study	No	6	Middle	(12), (27), (45), (48)
Zhang (36)	2025	China	Mean 38 ± 17 years (adult)	119	Single-center	Retrospective cohort study	Partial	8	High	(12), (19), (27), (49), (50), (51), (52)
Zhang (37)	2026	China	Mean 8.48 ± 4.89 years, range 2–14 years (pediatric)	40	Single-center	Retrospective cohort study	No	8	High	(13), (24), (53), (54), (55)
Zhang (38)	2020	China	18–75 years	265	Single-center	Retrospective cohort study	Partially	8	High	(27), (32), (42), (56), (57)
Zhou (39)	2025	China	Median 7.0 years	125	Multi-center	Retrospective cohort study	Yes	9	High	(1), (19), (50), (58)
Zhu (40)	2026	China	Median 41 years	118	Single-center	Retrospective cohort study	Partially	8	High	(12), (59)
Chen (41)	2024	China	≥16 years	91	Single-center	Retrospective cohort study	Yes	8	High	(19), (40), (67)
Liu (42)	2017	China	1.25–15 years	49	Single-center	Retrospective cohort study	Yes	8	High	(1)
Pei (43)	2015	China	1.5–13 years	24	Single-center	Retrospective cohort study	Yes	8	High	(12), (57)
Tan (44)	2022	China	≥18 years	202	Single-center	Retrospective cohort study	No	8	High	(12), (56), (57), (60), (61), (62)
Yang (45)	2023	China	Mean 42 ± 18 years	157	Single-center	Retrospective cohort study	Partially	9	High	(1), (12), (23), (24), (41), (42), (45), (63), (64)
Zhou (46)	2022	China	≥18 years	38	Single-center	Retrospective cohort study	Yes	9	High	(65), (66)

Candidate factors are denoted by the following reference numbers. (1) Age; (2) SOFA score, Sequential Organ Failure Assessment score; (3) Renal complications; (4) ECMO duration, Duration of ECMO support; (5) VIS before ECMO, Vasoactive-Inotropic Score before ECMO; (6) Duration from shock to ECMO, Duration from shock to ECMO initiation (hours); (7) Distal perfusion catheterization; (8) Treatment with IVIG, Treatment with intravenous immunoglobulin (IVIG); (9) Left ventricular dilatation after ECMO; (10) MODS, Multiorgan dysfunction syndrome; (11) Non-sinus rhythm; (12) LVEF, Left ventricular ejection fraction (LVEF); (13) VT/VF, Ventricular tachycardia/Ventricular fibrillation; (14) Severe histologic type, Severe histologic type in lymphocytic myocarditis; (15) Viral confirmed; (16) Charlson comorbidity index; (17) PEEP, Positive end-expiratory pressure at 24 h after ECMO; (18) Peri-ECMO RRT, Peri-ECMO renal replacement therapy; (19) Lactate; (20) CK-MB, Creatine kinase myocardial band; (21) Heart rate; (22) White blood cell count; (23) Total bilirubin; (24) Left ventricular end-systolic dimension; (25) ACEF score, Age-Creatinine-Ejection Fraction score; (26) Time from onset to admission; (27) Creatinine; (28) Tendency of hs-cTnI, Tendency of high-sensitivity cardiac troponin I; (29) pH, Arterial pH; (30) Cardiac arrest; (31) Atrioventricular block; (32) QRS prolongation; (33) CRP, C-reactive protein; (34) Peak hs-TnT during ICU, Peak high-sensitivity troponin T during intensive care unit stay; (35) Giant cell myocarditis; (36) Eosinophilic myocarditis; (37) Unspecified histopathology; (38) LT3S, Low triiodothyronine syndrome; (39) FT3, Free triiodothyronine; (40) SBP, Systolic blood pressure; (41) Peak NT-pro BNP, Peak N-terminal pro-brain natriuretic peptide; (42) Peak TnI, Peak troponin I; (43) CRRT, Continuous renal replacement therapy; (44) Female sex; (45) eGFR, Estimated glomerular filtration rate; (46) LA decompression, Left atrial (LA) decompression; (47) Myoglobin;(48) Left atrium dimension; (49) Respiratory symptoms; (50) CPR, Cardiopulmonary resuscitation; (51) Direct bilirubin; (52) TSH, Thyroid-stimulating hormone; (53) Hospital length of stay; (54) CK, Creatine kinase; (55) IL-6, Interleukin-6; (56) Cardiogenic shock; (57) BNP, Brain natriuretic peptide; (58) NPAR, Neutrophil percentage-to-albumin ratio; (59) 24-h urine volume; (60) Left ventricular fractional shortening; (61) Creatinine clearance rate; (62) Troponin T; (63) Complicated malignant arrhythmia; (64) Uric acid; (65) 24-h Cr decline rate, 24-hour creatinine decline rate; (66) 24-h CKMB decline rate, 24-hour creatine kinase myocardial band decline rate; (67) Chest pain.

Regarding ECMO support, ten studies (9, 23, 24, 26, 27, 39, 41–43, 46) included patients who all received ECMO, twelve studies (13, 28–34, 36, 38, 40, 45) included a subset of patients who received ECMO, and four studies (25, 35, 37, 44) either did not use ECMO or did not explicitly report ECMO use.

Methodological quality assessment indicated that 21 studies (13, 23–28, 30, 32, 33, 36–46) were of high quality (NOS score 7–9) and 5 studies (9, 29, 31, 34, 35) were of moderate quality (NOS score 5–6), justifying inclusion for quantitative meta-analysis.

### 3.3 Meta-analysis results

#### 3.3.1 Age

Four studies (23, 26, 31, 33) reported the association between age and in-hospital mortality in FM patients (*n* = 1,154). Between-study heterogeneity was low (*I*^2^ = 18%). Therefore, a fixed-effects model was used. Meta-analysis showed that age was independently associated with in-hospital mortality among FM patients (OR = 1.03, 95% CI: 1.02–1.04, *P* < 0.001). The forest plot is shown in Figure 2.

**Figure 2:**
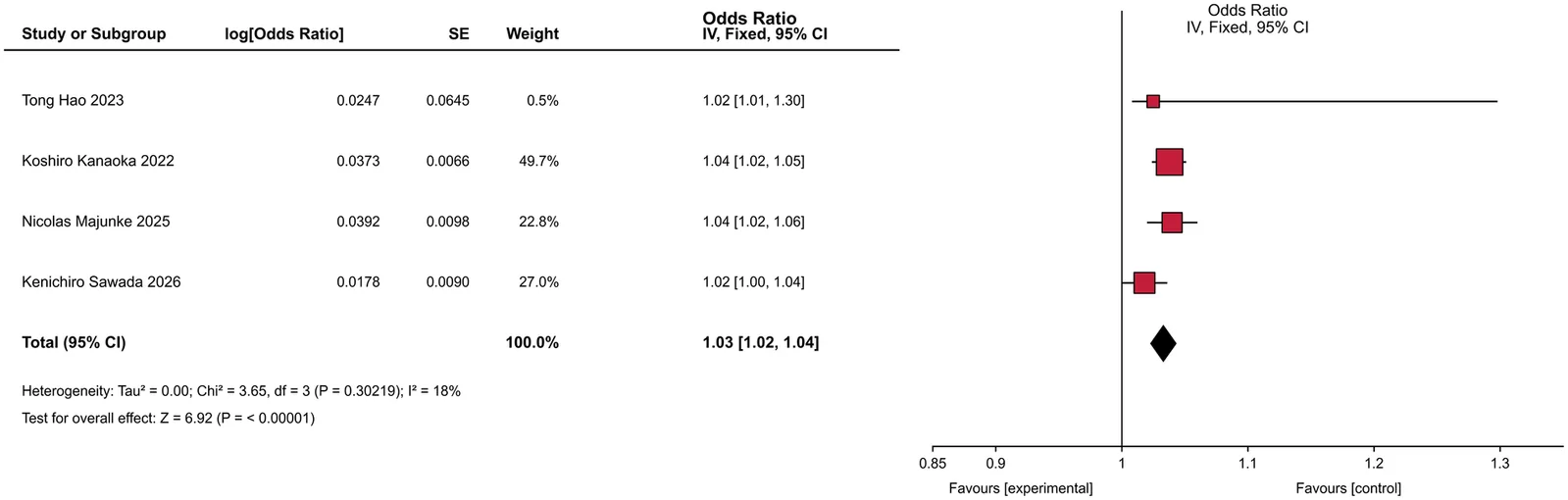
Forest plot of the meta-analysis for the association between age and in-hospital mortality in patients with FM.

#### 3.3.2 Cardiac arrest or cardiopulmonary resuscitation (CA/CPR)

Three studies (31, 36, 39) reported the association between CA/CPR and in-hospital mortality in FM patients (*n* = 515). Moderate heterogeneity was observed (*I*^2^ = 44%), and a fixed-effects model was applied. Pooled results indicated that CA/CPR was significantly associated with higher in-hospital mortality (OR = 2.57, 95% CI: 1.49–4.44, *P* = 0.0007). The forest plot is shown in Figure 3.

**Figure 3:**
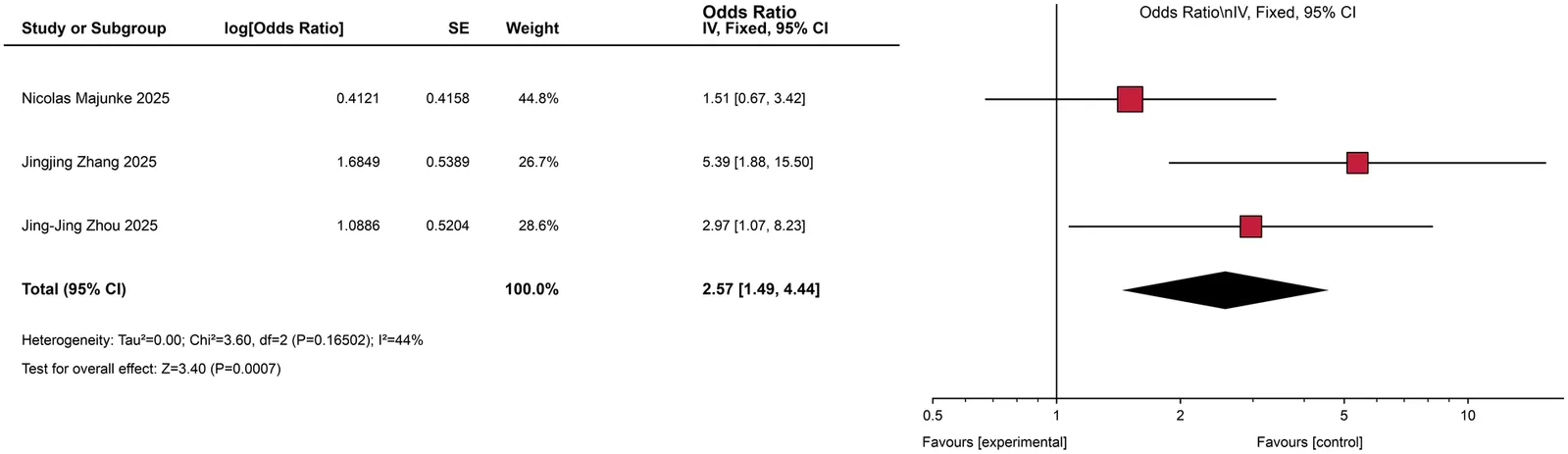
Forest plot of the meta-analysis for the association between CA/CPR and in-hospital mortality in patients with FM.

#### 3.3.3 Cardiac troponin I

Three studies (31, 32, 38) reported the association between cTnI levels and in-hospital mortality among FM patients (*n* = 632). No statistical heterogeneity was detected (*I*^2^ = 0%), and a fixed-effects model was used. Meta-analysis showed that elevated cTnI was independently associated with in-hospital mortality (OR = 1.04, 95% CI: 1.01–1.07, *P* = 0.0113). The forest plot is shown in Figure 4.

**Figure 4:**
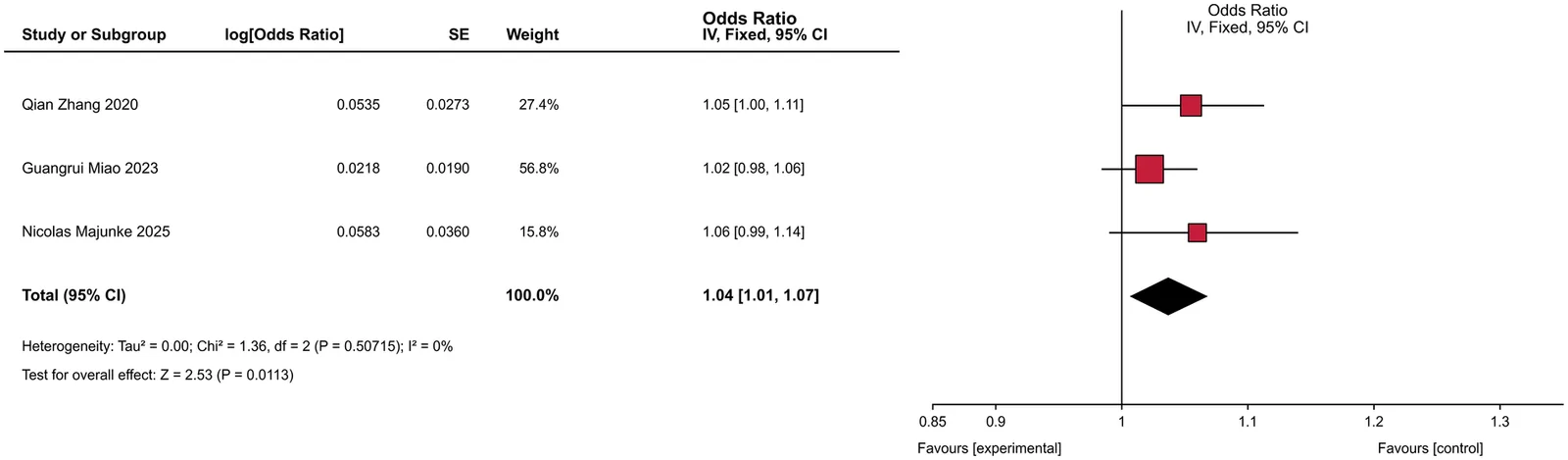
Forest plot of the meta-analysis for the association between cTnI and in-hospital mortality in patients with FM.

#### 3.3.4 Intravenous immunoglobulin therapy

Three studies (24, 25, 30) reported the association between intravenous immunoglobulin (IVIG) therapy and in-hospital mortality in FM patients (*n* = 258). Moderate heterogeneity was present (*I*^2^ = 50%), so a random-effects model was used. Pooled results showed an inverse association between IVIG therapy and in-hospital mortality (OR = 0.12, 95% CI: 0.02–0.86, *P* = 0.0347). The forest plot is shown in Figure 5.

**Figure 5:**
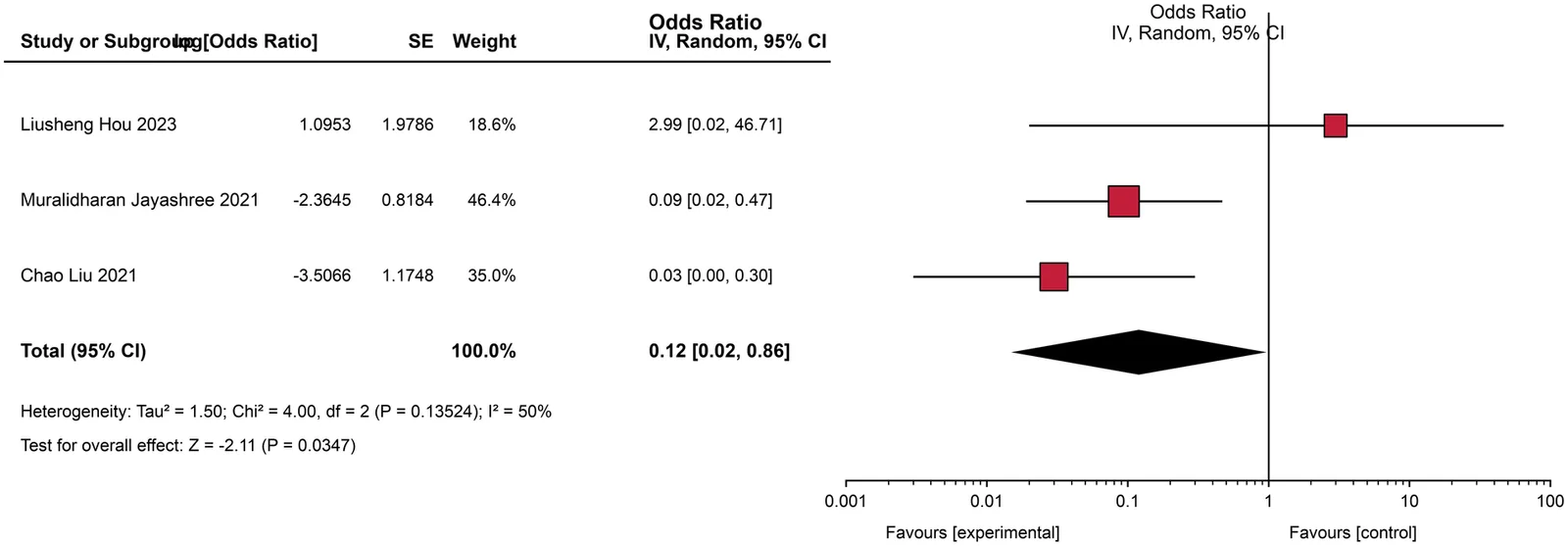
Forest plot of the meta-analysis for the association between IVIG therapy and in-hospital mortality in patients with FM.

#### 3.3.5 Lactate

Three studies (28, 36, 39) reported the association between serum lactate levels and in-hospital mortality in FM patients (*n* = 277). High heterogeneity was observed (*I*^2^ = 80%), and a random-effects model was applied. Pooled results indicated that elevated lactate was positively associated with in-hospital mortality (OR = 1.08, 95% CI: 1.00–1.16, *P* = 0.0467). The forest plot is shown in Figure 6.

**Figure 6:**
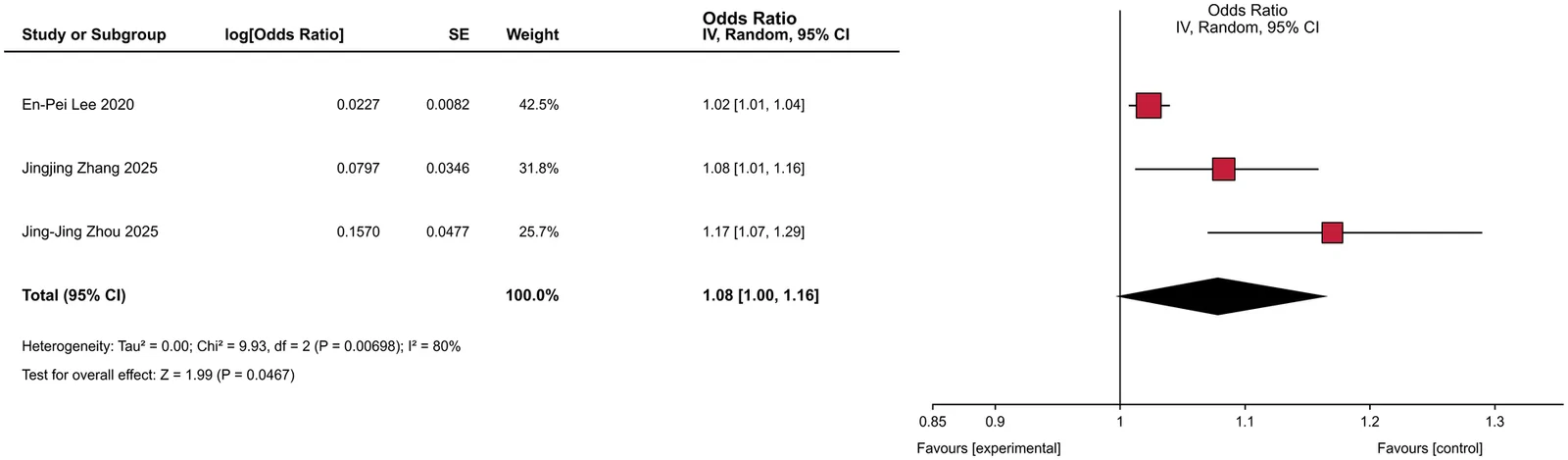
Forest plot of the meta-analysis for the association between serum lactate and in-hospital mortality in patients with FM.

#### 3.3.6 Left ventricular ejection fraction (LVEF)

Seven studies (28, 31, 32, 34, 36, 40, 44) reported the association between LVEF and in-hospital mortality among FM patients (*n* = 876). Heterogeneity was relatively high (*I*^2^ = 66%), and a random-effects model was used. Pooled results showed that reduced LVEF was significantly associated with higher in-hospital mortality (OR 0.94, 95% CI 0.90–0.97, *P* = 0.0014). The forest plot is shown in Figure 7.

**Figure 7:**
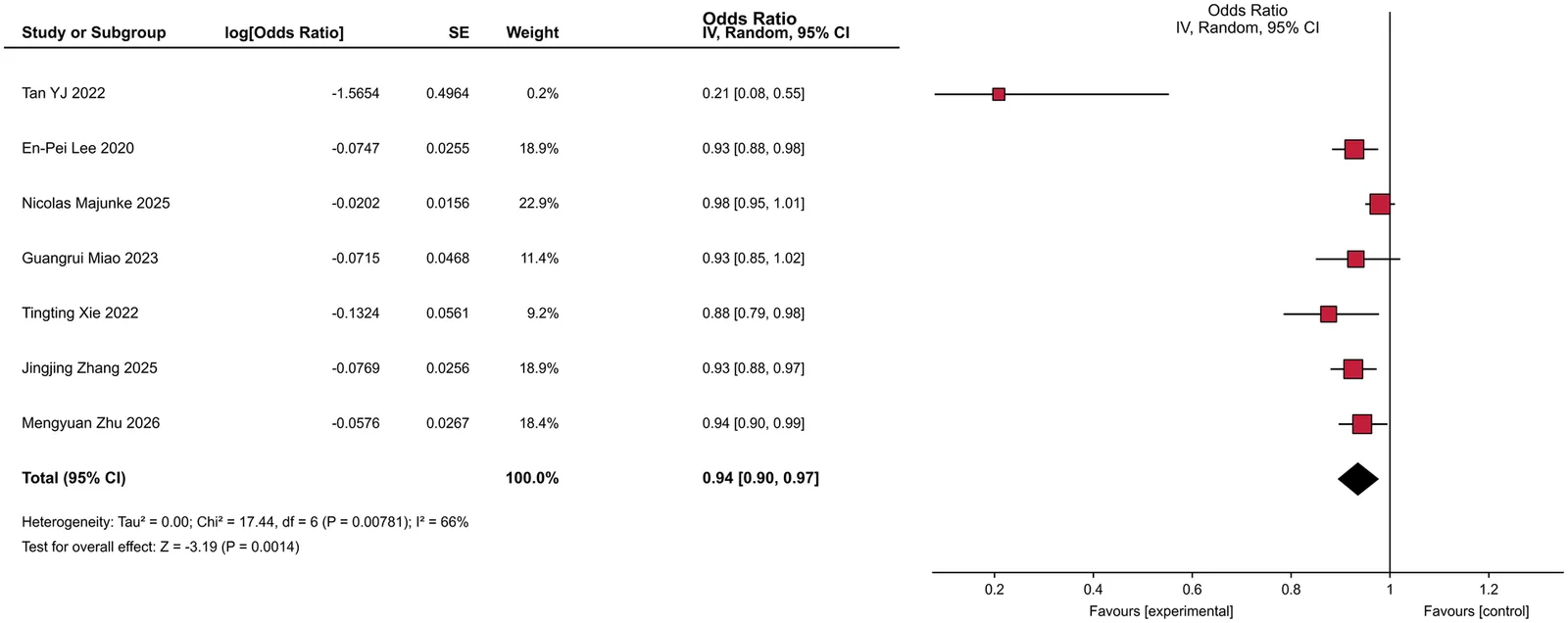
Forest plot of the meta-analysis for the association between LVEF and in-hospital mortality in patients with FM.

#### 3.3.7 Factors without statistical significance

Several clinical variables exhibited no significant pooled association with all-cause in-hospital mortality after meta-analytic synthesis, including creatine kinase myocardial band (CK-MB; 3 studies, *n* = 615), serum creatinine (4 studies, *n* = 647), total bilirubin (3 studies, *n* = 511), and ventricular tachycardia/ventricular fibrillation (VT/VF; 6 studies, *n* = 1,178) (all *P* > 0.05). Detailed results are presented in the Supplementary Figure S1.

### 3.4 Subgroup analysis

For cTnI, the association with in-hospital mortality remained statistically significant in the subgroup restricted to Chinese cohorts, consistent with the overall pooled result (Figure 8). Significant associations (*P* < 0.05) between reduced LVEF and elevated in-hospital mortality were observed across all three subgroups, including pediatric populations (Figure 9), Chinese cohorts (Figure 10), and patients receiving ECMO support (Figure 11). Subgroup analyses for other factors did not yield additional significant findings due to the limited number of studies available for stratification. All subgroup results should be interpreted as exploratory.

**Figure 8:**
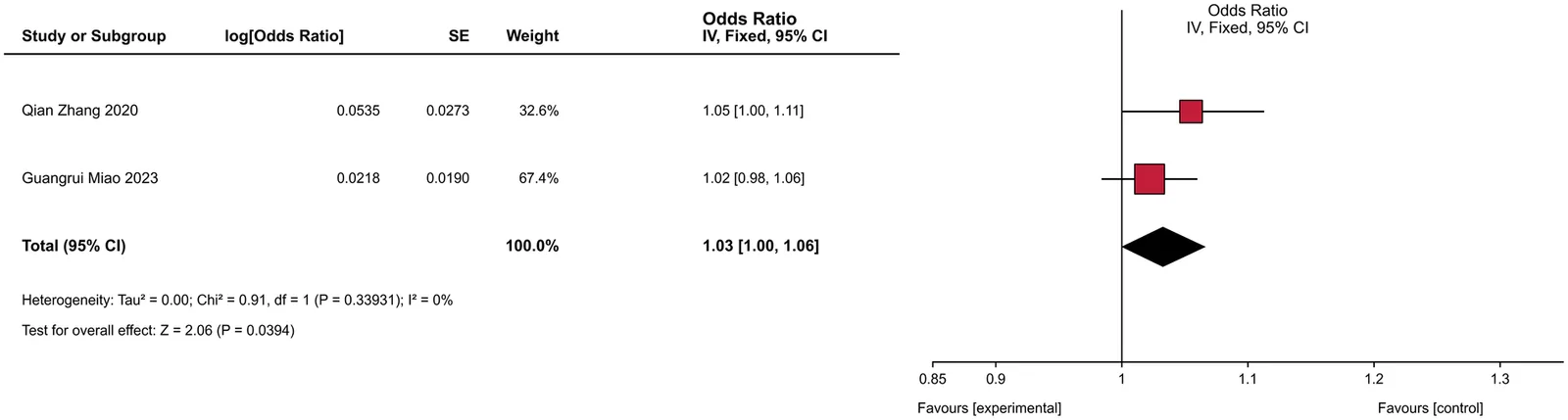
Subgroup-analysis forest plot for cTnI.

**Figure 9:**
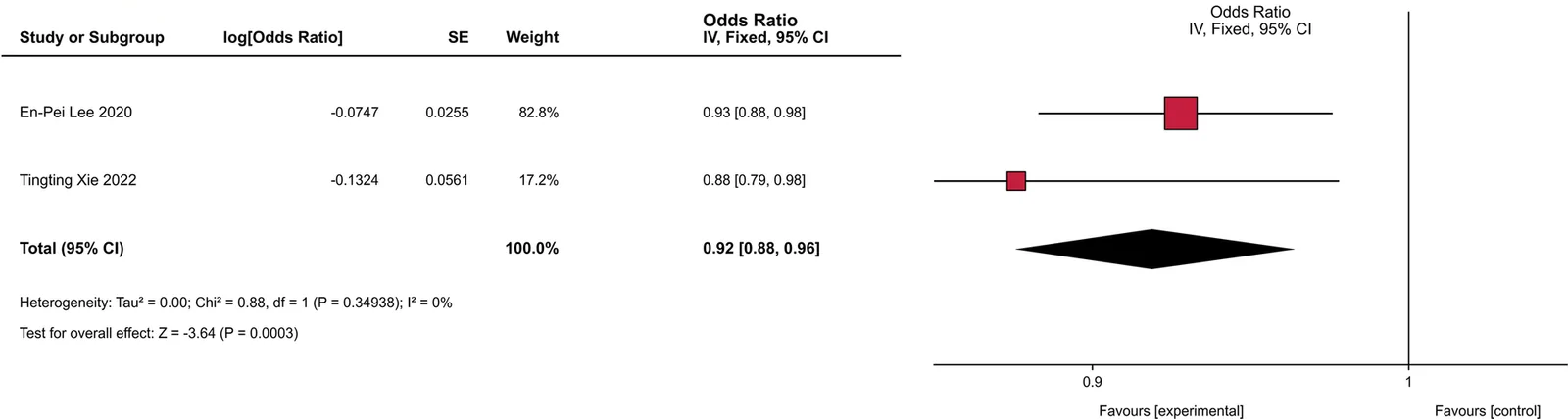
Subgroup-analysis forest plot for LVEF in pediatric populations.

**Figure 10:**
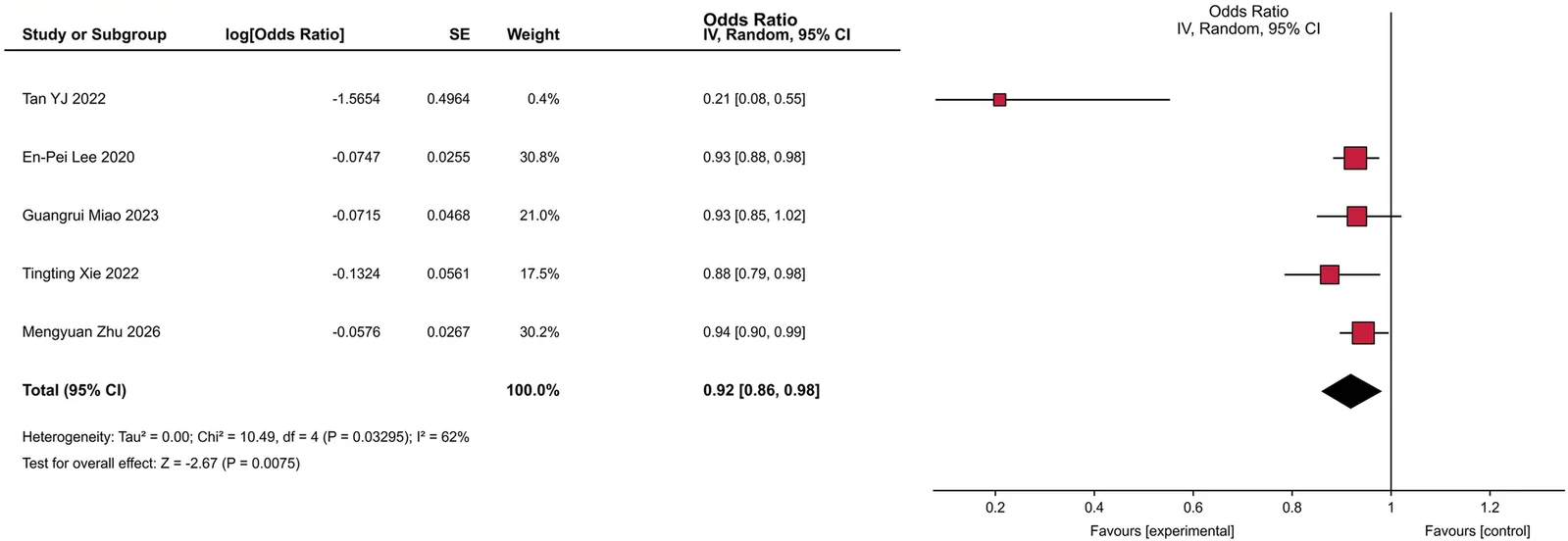
Subgroup-analysis forest plot for LVEF in Chinese cohorts.

**Figure 11:**
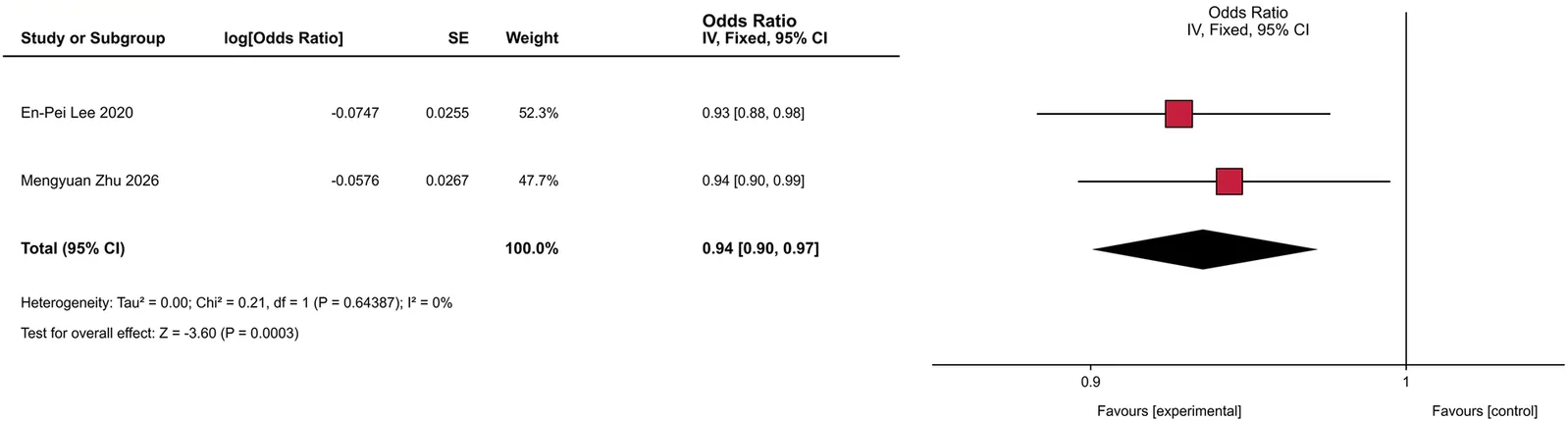
Subgroup-analysis forest plot for LVEF in patients receiving ECMO support.

### 3.5 Sensitivity analysis

Leave-one-out sensitivity analysis confirmed the robustness of the pooled estimates for age, cTnI, CA/CPR, and LVEF. The direction of effect remained unchanged, and all re-pooled results retained statistical significance after sequential removal of each individual study. For lactate, removal of a single study resulted in loss of statistical significance, indicating limited robustness of the pooled estimate. For IVIG, the pooled estimate was also unstable. One study showed an effect direction opposite to the others, and removal of this study eliminated the statistical significance of the overall association. Detailed results are presented in the Supplementary Figure S2.

### 3.6 Publication bias assessment

Funnel plots for factors with the largest numbers of contributing studies showed a generally symmetrical distribution of effect estimates, suggesting a low risk of publication bias. For factors with fewer than ten studies, statistical tests (Egger's and Begg's) were not performed due to insufficient power. Based on GRADE conservative evaluation principles, factors with fewer than five contributing studies were presumed to carry a potential risk of publication bias and were downgraded accordingly. Detailed results are presented in the Supplementary Figure S3.

### 3.7 GRADE evidence-certainty assessment

The GRADE-certainty ratings for the six statistically significant factors are summarized in Table 2. Age and cTnI were rated as low-certainty evidence. Lactate, LVEF, CA/CPR, and IVIG were rated as very-low-certainty evidence. Evidence downgrading was mainly driven by limited sample sizes, statistical imprecision, substantial between-study heterogeneity, and potential overall risk of bias. To enhance clinical interpretability, a comprehensive evidence-summary plot was generated to visualize five non-treatment-related prognostic associations with in-hospital mortality. IVIG was excluded from this plot given that its inverse association represented a treatment-related and statistically fragile signal instead of a stable mortality- associated biomarker (Figure 12).

**Table 2:** GRADE evidence-certainty assessment of significant factors associated with in-hospital mortality in FM patients.

Factor	Certainty assessment								Effect OR (95% CI)	*P* value	*I* ^2^	Certainty
	studies (*n*)	study design	Risk of bias	Inconsistency	Indirectness	Imprecision	Publication bias	Patients (*n*)				
Age	4	Retrospective cohort	Not serious	Not serious (*I*^2^ = 18%)	Not serious	Not serious	Not detected	1,154	1.03 (1.02–1.04)	< 0.0001	18%	●●○○ LOW
cTnI	3	Retrospective cohort	Not serious	Not serious (*I*^2^ = 0%)	Not serious	Not serious	Not detected	632	1.04 (1.01–1.07)	0.0113	0%	●●○○ LOW
IVIG	3	Retrospective cohort	Serious^a^	Serious (*I*^2^ = 50%)	Not serious	Not serious	Possible	258	0.12 (0.02–0.86)	0.0347	50%	●○○○ VERY LOW
Lactate	3	Retrospective cohort	Serious^b^	Very serious (*I*^2^ = 80%)	Not serious	Not serious	Possible	277	1.08 (1.00–1.16)	0.0467	80%	●○○○ VERY LOW
LVEF	7	Retrospective cohort	Not serious	Serious (*I*^2^ = 66%)	Not serious	Not serious	Possible	876	0.94 (0.90–0.97)	0.0014	66%	●○○○ VERY LOW
CA/CPR	3	Retrospective cohort	Not serious	Not serious (*I*^2^ = 44%)	Not serious	Serious^c^	Possible	515	2.57 (1.49–4.44)	0.0007	44%	●○○○ VERY LOW

RoB, risk of bias. All studies are retrospective cohort designs (Newcastle-Ottawa Scale score ≥6). GRADE starts at low-certainty for observational evidence.

a Potential confounding by indication and treatment selection bias.

b Potential measurement-timing bias related to shock stage, ECMO timing, and hemodynamic support.

c Wide confidence interval with small number of studies.

●●●● = HIGH; ●●●○ = MODERATE; ●●○○ = LOW; ●○○○ = VERY LOW.

**Figure 12:**
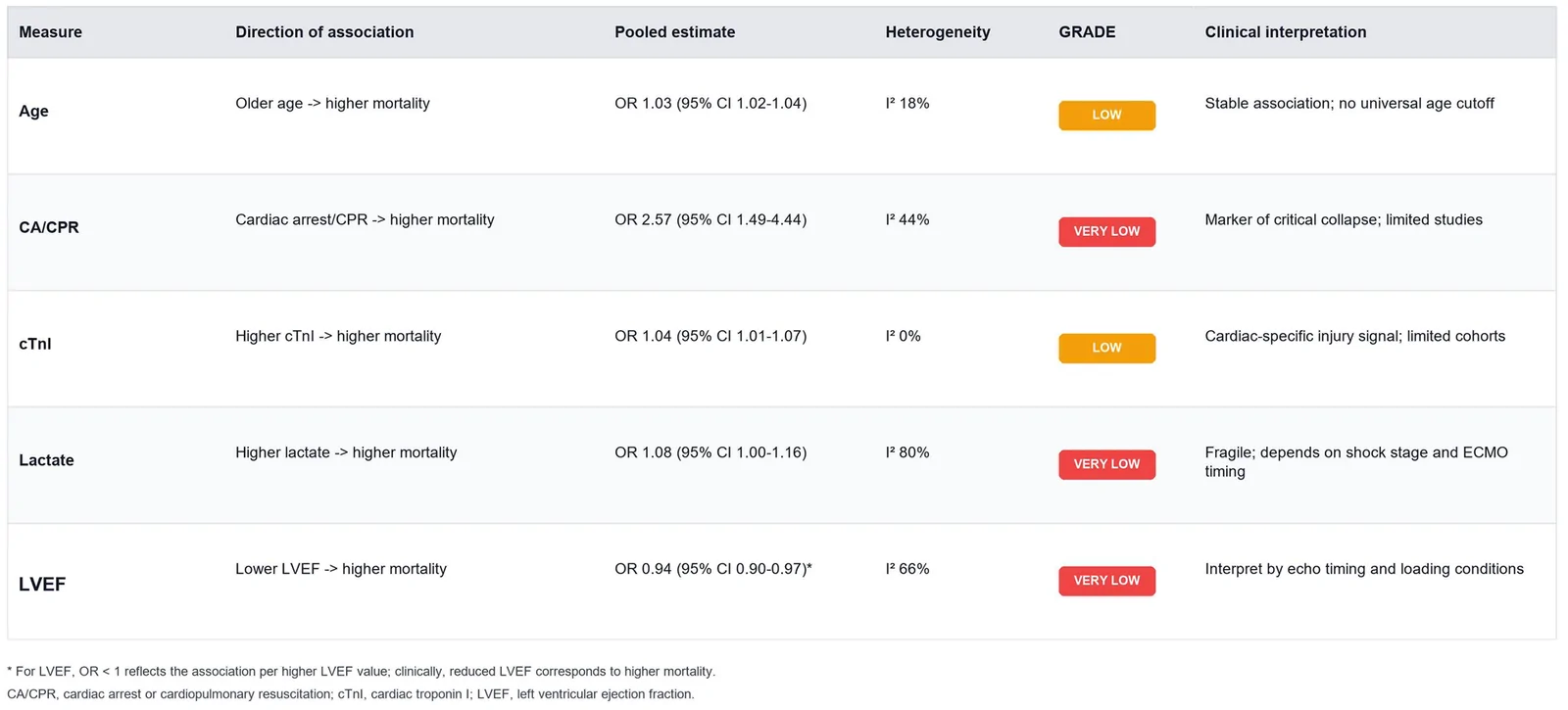
Evidence-summary plot of five prognostic indicators for all-cause in-hospital mortality in FM.

## 4 Discussion

### 4.1 Overall findings and academic value

This systematic review and meta-analysis synthesized data from global retrospective cohort studies to evaluate factors associated with in-hospital mortality in patients with FM. Published single-center studies show inconsistent associations between clinical variables and mortality outcomes, while prior evidence syntheses lack unified quantitative pooling of relevant associated factors and formal certainty appraisal to inform clinical risk stratification. In this work, 26 retrospective cohorts comprising 4,890 patients were pooled to quantify adjusted associations between candidate clinical and laboratory variables and in-hospital mortality. All available datasets were processed with prespecified subgroup and sensitivity analyses, and the GRADE framework was applied to assess the certainty of pooled effect estimates.

Quantitative synthesis identified four variables positively associated with elevated in-hospital mortality. LVEF showed an inverse statistical association, and lower LVEF values corresponded to higher mortality risk. One treatment-associated inverse signal for IVIG proved fragile per sensitivity analyses. Four additional biomarkers or clinical variables showed no statistically significant pooled associations after synthesis. This differentiation delivers aggregated clinical evidence to inform acute-phase risk-screening practice for FM.

The present work delivers two layers of incremental contributions to existing literature. Clinically, it systematically distinguishes mortality-associated clinical variables from markers more likely to reflect advanced multi-organ injury, addressing the long-standing inconsistency of findings across single-center reports. Methodologically, this analysis establishes a standardized quantitative synthesis pipeline paired with transparent GRADE evidence grading, a combination rarely implemented in previous relevant summaries. The unified analytical framework presented here can serve as a reference for future prospective multicenter investigations aiming to reinforce the evidence base for FM prognostic assessment.

### 4.2 Mechanisms, between-study heterogeneity and clinical implications of significant mortality-associated factors

#### 4.2.1 Age

Consistent with pooled effect estimates derived from multivariable-adjusted data of included cohorts, age showed an independent prognostic association with in-hospital mortality, which persisted after adjustment for multiple confounders (10). Existing literature reports divergent mortality patterns across distinct age subgroups (47, 48). Adult populations exhibit a linear positive association between advancing age and short-term in-hospital mortality, whereas studies focused solely on pediatric cohorts report sharply elevated mortality risk among infants younger than two years (49).

All age-related effect sizes pooled in this analysis were extracted from primary studies using multivariate regression models adjusting for baseline comorbidities and acute cardiogenic shock. These adjustments partially mitigate confounding influences from these core clinical variables, yet residual unmeasured confounders cannot be fully excluded. The excess mortality risk observed among infants aligns with biologically plausibility (50). Immature immune regulatory systems may be accompanied by severe inflammatory storms, a state frequently observed alongside irreversible circulatory failure (51).

Predefined subgroup stratification contrasting pediatric and adult patients precluded quantitative comparison of age-related effect magnitudes, due to two objective constraints. Only four eligible studies provided extractable age-associated effect sizes, and few original publications reported uniformly stratified effect estimates separated by age subgroups. Differences in disease triggers, inflammatory activation pathways and circulatory-support regimens between children and adults further complicate cross-population quantitative comparison (2). Notably, the low between-study heterogeneity of the pooled age association indicates the overall positive association remains stable despite disparate subgroup risk trends.

Clinically, chronological age can serve as a preliminary reference indicator for acute-phase risk assessment upon admission. Nevertheless, population-specific risk cutoff values cannot be recommended based on current evidence. Combined with the low GRADE certainty rating for age-related evidence, future prospectively designed multicenter cohorts with standardized stratified sampling and unified data-recording protocols are required to generate age-stratified effect estimates for reference in future risk threshold development.

#### 4.2.2 Cardiac arrest or cardiopulmonary resuscitation

Among all mortality-associated factors identified in this meta-analysis, CA/CPR after disease onset showed the strongest association with in-hospital mortality. This event occurs in the setting of extensive myocardial injury and irreversible hemodynamic collapse. Even with timely cardiopulmonary resuscitation and ECMO-assisted circulatory support, patients remain susceptible to cerebral hypoperfusion and progressive multiple-organ failure, conditions that are observed alongside higher in-hospital mortality (52).

Consistent findings from previous cohorts confirm that cardiac arrest signals critical disease status, yet unified protocols for early risk screening and preemptive supportive measures have not been widely adopted in routine clinical practice (53). Pooled estimates demonstrate a strong association between circulatory arrest and elevated in-hospital mortality, while findings from the FULMOON cohort study show that timely circulatory-support deployment among patients with refractory shock or frequent malignant arrhythmias was observed alongside reduced subsequent cardiac arrest (10), with corresponding favorable outcomes reported in that cohort (54). Nevertheless, given the very-low GRADE-certainty evidence for the pooled effect sizes in the meta-analysis, the stratified alert strategy derived from the above evidence can only serve as a reference rather than a mandatory standard for FM management across intensive-care units. Well-designed prospective studies are still required to validate this intervention pathway before wide-scale clinical application.

#### 4.2.3 Lactate

Elevated serum lactate was positively associated with in-hospital mortality, yet the pooled estimate was accompanied by substantial between-study heterogeneity and limited robustness in sensitivity analysis. This cross-cohort discrepancy can be interpreted from both pathogenetic and methodological perspectives. From a mechanistic perspective, lactate dynamics are associated with the progression of cardiogenic shock, and blood collection timing exerts a decisive influence on result interpretation (55). Lactate concentrations rise mildly during early tissue hypoperfusion and only peak when multi-organ circulatory insufficiency emerges in advanced shock stages (56).

Substantial inconsistencies in blood sampling timing across included cohorts constitute a primary source of heterogeneity, with measurements collected at admission, mid-shock course or post-ECMO initiation lacking direct comparability. Variations in ECMO adoption further widen inter-cohort gaps. Extracorporeal circulation rapidly restores tissue perfusion and reduces circulating lactate concentrations (57, 58). Accordingly, cohorts with higher ECMO utilization tend to adopt lower lactate thresholds for risk assessment. Additionally, inconsistent covariate adjustment strategies across regression models further contribute to cross-study variation. Leave-one-out sensitivity analysis confirmed that the pooled association was largely driven by a single cohort, indicating unstable overall effect estimation.

Mechanistically, sustained hyperlactatemia may reflect severe myocardial energy-metabolism disorder and accelerating multi-organ injury, clinical manifestations observed alongside higher mortality risk even after adjustment for shock status and circulatory support in primary studies (56). Clinically, universal lactate cutoffs derived from single-center datasets cannot be directly generalized to other medical institutions. Both sampling time and whether patients receive ECMO support should be integrated into comprehensive severity evaluation.

Notably, lactate data were extracted from merely three cohorts enrolling 277 patients in total, and the corresponding evidence was rated very-low certainty per GRADE assessment. The fragile pooled effect and its sensitivity to single-study removal restrict broad clinical promotion of unified lactate thresholds. These findings support interpreting lactate according to sampling time, shock stage, and ECMO status rather than applying a universal threshold across cohorts.

#### 4.2.4 Cardiac troponin I

Elevated cTnI showed an independent prognostic association with in-hospital mortality, and the pooled analysis detected zero between-study heterogeneity across eligible cohorts. Subgroup analyses further validated significant associations within Chinese patient subgroups, though stratified comparisons across pediatric and adult populations did not yield consistent statistical signals. This biomarker exhibits distinct mortality-associated associations relative to CK-MB, a discrepancy largely attributable to superior myocardial tissue specificity of cTnI (59). Unlike CK-MB, cTnI measurement is unaffected by skeletal muscle injury or systemic inflammatory responses, which reduces non-cardiac interference in risk evaluation (60). Uniform detection assays, unified cutoff definitions and identical variable transformation protocols adopted by all primary studies collectively account for the complete consistency of associative effects observed in this meta-analysis.

Mechanistically, sustained high cTnI concentrations correspond to extensive cardiomyocyte necrosis, a state observed alongside impairs LVEF, cardiogenic shock and malignant arrhythmia, all clinical manifestations frequently seen among non-survivors (12). The zero inter-cohort heterogeneity highlights the robust stability of this association when biomarker testing is standardized across medical centers. From a clinical perspective, cTnI is suited to serve as the core laboratory indicator for quantifying myocardial-injury severity during the acute phase of FM. Compared with CK-MB, cTnI delivers more reliable risk signals free from extra-cardiac confounding factors, which may serve as a reference to streamline acute-phase risk evaluation in subsequent prospective validation studies (60).

It should be noted that relevant data were extracted from only three cohorts comprising 632 participants, and the GRADE framework rated this body of evidence as low certainty. Although cTnI possesses evident laboratory advantages, universal replacement of CK-MB in all clinical settings cannot be fully recommended based on limited retrospective evidence. Standardized multi-center prospective registrations are still required to verify whether cTnI can independently simplify routine myocardial-injury screening across diverse patient populations.

#### 4.2.5 Left ventricular ejection fraction

Reduced LVEF was associated with in-hospital mortality, with an *I*^2^ value of 66% indicating high cross-cohort heterogeneity. A primary source of this inconsistency lies in divergent timing of echocardiographic acquisition across participating cohorts. Extracorporeal circulation exerts direct impacts on ventricular preload and afterload (61). Echocardiograms taken after ECMO initiation cannot be directly compared against admission or pre-ECMO baseline scans, and such altered loading conditions may distort LVEF readings (62). Beyond variable imaging timing, inter-cohort discrepancies stem from heterogeneous prevalence of pre-existing chronic heart disease at enrolment, alongside inconsistent adjustment of vasoactive medication duration and shock persistence within multivariable regression frameworks.

Subgroup stratification yielded robust associative signals only among pediatric and Chinese patient cohorts, while stratified effect sizes for other populations could not be reliably synthesized due to insufficient stratified raw data released by primary studies. Unlike age-related analyses which mainly suffered from inconsistent age-threshold reporting, LVEF heterogeneity originates uniquely from echocardiogram-timing bias and ECMO-mediated hemodynamic changes. These factors create distinct obstacles to forming universal risk cutoffs applicable across all medical settings.

Mechanistically, sustained left ventricular systolic impairment coincides with diminished systemic cardiac output and protracted whole-body tissue hypoperfusion, clinical features observed alongside intractable cardiogenic shock and subsequent in-hospital mortality (5). Even after adjustment for ECMO intervention and baseline cardiac history in multivariable models from primary studies, LVEF remained associated with mortality, suggesting that early systolic dysfunction may retain prognostic information beyond transient imaging artefacts induced by mechanical support. Clinically, initial bedside echocardiography measuring LVEF can support rapid screening and offer suggestive prognostic clues to identify patients who may require early intensive circulatory support, whereas serial echocardiograms obtained after ECMO initiation should be interpreted according to contemporaneous loading conditions and are not directly interchangeable with baseline scans for initial acute-risk stratification.

All LVEF-related pooled data were aggregated from seven retrospective cohorts enrolling a total of 876 participants, and GRADE assessment classified this body of evidence as very-low certainty. Noticeable imaging-derived heterogeneity and limited stratified data restrict the derivation of population-specific LVEF risk thresholds. Therefore, LVEF should be interpreted according to the timing of echocardiography and concurrent loading conditions, particularly in patients receiving ECMO or other temporary mechanical circulatory support.

#### 4.2.6 Intravenous immunoglobulin therapy

An inverse association between IVIG therapy and in-hospital mortality was derived from multivariable-adjusted effect estimates of primary studies, though this association signal was graded as very-low-certainty evidence by the GRADE framework. Only three retrospective cohorts enrolling a total of 258 participants were eligible for quantitative pooling, with moderate between-study heterogeneity. The most prominent source of cross-cohort inconsistency stems from severe indication confounding present across the included datasets. Patients without refractory cardiogenic shock and with milder multi-organ injury were preferentially administered IVIG therapy (63). Conversely, patients with catastrophic shock or immediate cardiac arrest may have been triaged directly to mechanical circulatory support, thereby reducing their opportunity to receive or complete IVIG therapy. This inherent treatment-selection bias driven by baseline disease severity may overestimate the apparent inverse association linked to IVIG therapy (64).

Findings from basic mechanistic studies report that IVIG may be accompanied by neutralization of pathogenic autoantibodies (64) and suppression of excessive myocardial inflammatory cascades, observations offering theoretical plausibility for the inverse association seen in the present meta-analysis among FM patients. However, such theoretical immunomodulatory properties cannot be reliably validated by the unstable pooled results and should not be translated into a causal treatment claim. Leave-one-out sensitivity analysis further verified the fragility of this associative estimate. The 2023 cohort reported by Hou et al. (24) demonstrated an effect direction opposite to the other two included studies. Exclusion of this single outlier completely eliminated the statistical significance of the overall pooled association, indicating the observed survival-related signal was heavily driven by limited study data. Distinct protocols for IVIG dosage and timing adopted in Hou's cohort may partially explain its divergent findings relative to the other enrolled research.

No universal consensus has been reached among current clinical guidelines on routine IVIG administration for all FM patients, as existing clinical evidence remains fragmented and underpowered. Given the very-low-certainty evidence and prominent treatment-selection bias identified in this meta-analysis, standardized widespread IVIG application cannot be recommended across the full FM population. The observed inverse association may partly arise from bias toward clinically more stable FM patients who were able to receive IVIG. These limitations preclude definitive individualized medication guidance even after balancing potential therapeutic benefits and short-term immune-related adverse events. For critically ill patients complicated with refractory cardiogenic shock, insufficient quantitative pooled evidence is available to support routine IVIG intervention, and targeted prospective investigations remain necessary for this patient subgroup.

### 4.3 Interpretation of non-significant results

This meta-analysis identified no independent association between CK-MB, VT/VF, serum creatinine or total bilirubin and all-cause in-hospital mortality. Such neutral results arise from a combination of varied pathophysiological characteristics, cross-cohort methodological discrepancies, inconsistent clinical-intervention protocols and limited statistical power. These factors allow differentiation between intrinsic biomarker limitations and study-derived confounding effects.

The divergent prognostic associations of cTnI and CK-MB largely originates from differences in myocardial specificity. CK-MB concentrations may show falsely elevated values in the setting of systemic inflammation, skeletal muscle injury and invasive procedures, and cannot precisely quantify cardiomyocyte necrosis (65). In addition, inconsistent dichotomous or continuous transformation standards for CK-MB across cohorts weaken its pooled association. In contrast, unified detection protocols for cTnI yield zero between-study heterogeneity in the corresponding meta-analysis. The consistent independent prognostic association of cTnI, together with its superior myocardial specificity documented in primary studies, suggests cTnI may be better-suited for evaluating myocardial injury relative to CK-MB. Prospective diagnostic validation analyses are still required to confirm such comparative superiority.

No independent mortality-related association was observed for VT/VF, a finding largely attributable to heterogeneous timing of arrhythmia onset and variable rescue efficacy. Early ventricular arrhythmia may be rapidly controlled alongside defibrillation, antiarrhythmic drugs or ECMO support (66), whereas late arrhythmia occurring at terminal shock coincides with irreversible circulatory dysfunction (67). Varying extents of myocardial injury are observed alongside distinct arrhythmia phenotypes. Sustained ventricular fibrillation corresponds to massive cardiomyocyte loss (68). Prompt defibrillation combined with mechanical circulatory support may be accompanied by rapid restoration of systemic tissue perfusion (69), a state observed alongside offset adverse prognostic impacts of arrhythmic events (70). Coupled with limited case numbers of severe electrical storm in included cohorts, arrhythmia episodes should be regarded as an emergency-intervention trigger rather than standalone mortality risk markers.

Serum creatinine and total bilirubin showed no independent prognostic association, although their underlying pathological backgrounds differ substantially. Creatinine elevation may coincide with renal hypoperfusion observed among patients with cardiogenic shock (71). Its measurement is confounded by pre-existing chronic kidney disease, variable timing of CRRT initiation and vasoactive-agent doses (72). Renal dysfunction represents a delayed complication of circulatory collapse, and creatinine levels rise substantially later than myocardial-injury biomarkers (73). Further delays in renal biomarker elevation impair early risk discrimination (74). Hyperbilirubinemia may occur in the setting of cardiac hepatic congestion and inflammatory hepatocyte damage, with peak concentration appearing after elevation of myocardial-injury indicators (75). ECMO-assisted circulation and hepatoprotective interventions may be accompanied by improved hepatic perfusion and reduced bilirubin concentrations (76). Heterogeneous timing for implementation of such therapies creates prominent cross-cohort comparability barriers. Both organ-function markers reflect advanced multi-organ damage and are unsuitable for early acute-risk stratification.

Collectively, these non-significant biomarkers cannot serve as core standalone indicators to estimate in-hospital mortality risk, yet they retain auxiliary value for evaluating advanced organ injury. Contrasting the predictive limitations of these four markers against the stable mortality-related associations described above supports construction of a multi-layered assessment framework for acute-phase risk stratification among patients with FM.

### 4.4 Limitations and future directions

Several notable limitations of this analysis should be acknowledged. First, all included data were retrieved from retrospective cohort studies, which are inherently susceptible to selection bias and incomplete clinical documentation. Several key mortality-associated variables were derived from small cohorts with limited sample sizes, restricting overall statistical power and introducing substantial between-study heterogeneity across pooled estimates. Second, primary studies lacked unified standardized protocols for baseline clinical testing, echocardiographic acquisition, and multivariate confounding adjustment. Heterogeneous clinical measurement timings and inconsistent adjustment strategies across studies inevitably introduced systematic discrepancies, even after exclusive synthesis of fully adjusted effect sizes. Third, analyses of several non-significant biomarkers partially relied on unadjusted univariate data from original studies. Residual confounding could not be completely ruled out, leaving their independent prognostic association with in-hospital mortality unconfirmed. Fourth, stratified analyses across multiple clinical dimensions were unfeasible due to insufficient extractable stratified raw data. Formal publication bias tests were not performed owing to the limited number of included studies per outcome. Although funnel plots showed symmetric distribution, potential unpublished small-sample negative studies could not be excluded, leading to GRADE evidence certainty downgrading. Furthermore, this review only included English and Chinese publications, possibly introducing language-based selection bias.

Future research directions are proposed to address the above evidence gaps. Large prospective multicenter registries covering both pediatric and adult populations are needed to quantify stratified prognostic associations across variable mechanical circulatory support and immunotherapy strategies. Standardized early baseline assessment windows should be established for unified prospective data collection. Core clinical variables should be systematically documented with uniform definitions to reduce measurement heterogeneity. For ECMO recipients, future studies should standardize the timing of laboratory and echocardiographic measurements relative to cannulation to minimize time-dependent measurement distortion. For immunotherapy regimens such as IVIG, prospective standardized recording of treatment characteristics and baseline disease profiles is warranted. Rigorous controlled or propensity-score-matched designs are required to mitigate inherent selection bias and further validate these prognostic associations. The mortality-associated variables identified in this analysis may serve as candidate predictors for future externally validated risk prediction models for acute FM. Subsequent systematic reviews should expand retrieval coverage to grey literature to reduce publication bias and enhance overall evidence robustness.

## 5 Conclusion

This meta-analysis quantitatively synthesized multiple prognostic associations with all-cause in-hospital mortality among patients with FM and evaluated evidence certainty using the GRADE framework. Based on multivariable-adjusted effect estimates from primary studies, advancing age, cardiac arrest or CPR, elevated lactate and increased cTnI were positively associated with higher in-hospital mortality. LVEF showed an inverse prognostic association with short-term survival. The inverse association between IVIG and mortality is statistically fragile and heavily influenced by indication-related and baseline-severity bias. Accordingly, these data cannot support an inference of true therapeutic benefit from IVIG.

Integrated evidence interpretation indicates that cTnI demonstrates more stable mortality-associated associations with acute myocardial injury than CK-MB under standardized detection conditions, though prospective validation is still required to confirm its clinical value as a preferred biomarker of myocardial injury. Arrhythmic episodes and conventional organ functional markers mainly reflect advanced physiological deterioration or urgent intervention requirements, rather than providing reliable standalone prognostic information. GRADE assessment demonstrated low-certainty evidence for age and cTnI, while all other significant associations were rated very-low certainty, attributable to limited sample sizes and prominent between-study heterogeneity.

This analysis provides a rigorous quantitative synthesis that resolves conflicting evidence from prior reviews and establishes comprehensive prognostic association profiles for acute FM. To further consolidate and translate these preliminary findings into clinical risk-stratification strategies, large-scale stratified prospective multicenter data are warranted to validate current associations and support future construction of standardized multi-marker evaluation systems.

## Data Availability

The original contributions presented in the study are included in the article/Supplementary Material, further inquiries can be directed to the corresponding author/s.
