# Supplementary material for: Associated factors for in-hospital mortality in patients with fulminant myocarditis: a systematic review and meta-analysis
Source: Front Cardiovasc Med. 2026 Sep 28;13:1938794. doi: 10.3389/fcvm.2026.1938794 (PMC13655156; doi:10.3389/fcvm.2026.1938794)
Supplement: Supplementary file 1 [file Datasheet1.docx]

Supplementary Table S1 Complete search strategy for PubMed database

| #1 | Search: Fulminant Myocarditis OR Acute Fulminant Myocarditis OR Fulminant viral myocarditis OR Severe Myocarditis[Title/Abstract] Sort by: Publication Date |
| --- | --- |
| #2 | Search: (Myocarditis[MeSH Terms]) OR (Myocarditides[Title/Abstract] OR Carditis[Title/Abstract] OR Myocarditis[Title/Abstract]) Sort by: Publication Date |
| #3 | #1 OR #2 |
| #4 | Search: In hospital Mortality OR Hospital Mortality OR In hospital Death OR All-cause Mortality OR Adverse Outcome OR Major Adverse Cardiovascular Events OR MACE OR Poor Prognosis[Title/Abstract] Sort by: Publication Date |
| #5 | Search: (Mortality[MeSH Terms]) OR (Mortalities[Title/Abstract] OR Mortality Rate[Title/Abstract] OR Mortality Rates[Title/Abstract] OR Rate, Mortality[Title/Abstract] OR Death Rate[Title/Abstract] OR Death Rates[Title/Abstract] OR Rate, Death[Title/Abstract] OR Mortality, Differential[Title/Abstract] OR Differential Mortality[Title/Abstract] OR Differential Mortalities[Title/Abstract] OR Mortality, Excess[Title/Abstract] OR Excess Mortality[Title/Abstract] OR Excess Mortalities[Title/Abstract] OR Mortality Determinants[Title/Abstract] OR Determinants, Mortality[Title/Abstract] OR Determinant, Mortality[Title/Abstract] OR Mortality Determinant[Title/Abstract] OR Case Fatality Rate[Title/Abstract] OR Case Fatality Rates[Title/Abstract] OR Rate, Case Fatality[Title/Abstract] OR Rates, Case Fatality[Title/Abstract] OR CFR Case Fatality Rate[Title/Abstract] OR Decline, Mortality[Title/Abstract] OR Mortality Declines[Title/Abstract] OR Mortality Decline[Title/Abstract] OR Age-Specific Death Rate[Title/Abstract] OR Age-Specific Death Rates[Title/Abstract] OR Death Rate, Age-Specific[Title/Abstract] OR Rate, Age-Specific Death[Title/Abstract] OR Age Specific Death Rate[Title/Abstract] OR Crude Death Rate[Title/Abstract] OR Crude Death Rates[Title/Abstract] OR Death Rate, Crude[Title/Abstract] OR Rate, Crude Death[Title/Abstract] OR Crude Mortality Rate[Title/Abstract] OR Crude Mortality Rates[Title/Abstract] OR Mortality Rate, Crude[Title/Abstract] OR Rate, Crude Mortality[Title/Abstract] OR Mortality[Title/Abstract]) Sort by: Publication Date |
| #6 | Search: (Death[MeSH Terms]) OR (End Of Life[Title/Abstract] OR End-Of-Life[Title/Abstract] OR Determination of Death[Title/Abstract] OR Near-Death Experience[Title/Abstract] OR Cardiac Death[Title/Abstract] OR Death, Cardiac[Title/Abstract] OR Death[Title/Abstract]) Sort by: Publication Date |
| #7 | #4 OR #5 OR #6 |
| #8 | Search: Influencing Factors OR Predictors OR Correlates[Title/Abstract] Sort by: Publication Date |
| #9 | Search: (Risk Factors[MeSH Terms]) OR (Factor, Risk[Title/Abstract] OR Risk Factor[Title/Abstract] OR Population at Risk[Title/Abstract] OR Populations at Risk[Title/Abstract] OR Risk Scores[Title/Abstract] OR Risk Score[Title/Abstract] OR Score, Risk[Title/Abstract] OR Risk Factor Scores[Title/Abstract] OR Risk Factor Score[Title/Abstract] OR Score, Risk Factor[Title/Abstract] OR Health Correlates[Title/Abstract] OR Correlates, Health[Title/Abstract] OR Social Risk Factors[Title/Abstract] OR Factor, Social Risk[Title/Abstract] OR Factors, Social Risk[Title/Abstract] OR Risk Factor, Social[Title/Abstract] OR Risk Factors, Social[Title/Abstract] OR Social Risk Factor[Title/Abstract] OR Risk Factors[Title/Abstract]) Sort by: Publication Date |
| #10 | #8 OR #9 |
| #11 | #3 AND #7 AND #10 |

Supplementary Figure S1 Forest plots of meta-analysis for four non-significant laboratory factors of in-hospital mortality

(A)Forest Plot for CK-Band; (B) Forest Plot for Serum Creatinine; (C) Forest Plot for Total Bilirubin; (D) Forest Plot for VT/VF.

Supplementary Figure S2 Leave-one-out sensitivity analysis forest plots for all six significant factors

(A‑D) Sensitivity analysis results for age; (E‑G) Sensitivity analysis for cTnI; (H‑J) Sensitivity analysis for IVIG; (K‑M) Sensitivity analysis for lactate; (N‑T) Sensitivity analysis for LVEF; (U‑W) Sensitivity analysis for CA/CPR.

Supplementary Figure S3 Funnel plots for publication bias assessment of six significant factors
